# AGS Gastric Cells: Antioxidant Activity and Metabolic Effects of Phenolic Extracts from Different Monocultivar Virgin Olive Oils

**DOI:** 10.3390/antiox12071347

**Published:** 2023-06-27

**Authors:** Paola Faraoni, Maria Bellumori, Lorenzo Cecchi, Beatrice Zonfrillo, Marzia Innocenti, Alessio Gnerucci, Nadia Mulinacci, Francesco Ranaldi

**Affiliations:** 1 Department of Experimental and Clinic Biomedical Sciences “Mario Serio”, University of Florence, Viale Pieraccini 6, 50139 Florence, FI, Italy; paola.faraoni@unifi.it (P.F.); francesco.ranaldi@unifi.it (F.R.); 2 Department of NEUROFARBA, Division of Pharmaceutical and Nutraceutical Sciences, University of Florence, Via U. Schiff 6, 50019 Sesto Fiorentino, FI, Italy; maria.bellumori@unifi.it (M.B.); beatrice.zonfrillo@unifi.it (B.Z.); marzia.innocenti@unifi.it (M.I.); nadia.mulinacci@unifi.it (N.M.); 3 Department of Agricultural, Food and Forestry Systems Management (DAGRI), University of Florence, Piazzale Delle Cascine 16, 50144 Florence, FI, Italy; 4 Department of Physics and Astronomy, University of Florence, Via Sansone, 1, 50019 Sesto Fiorentino, FI, Italy

**Keywords:** AGS cells, extra virgin olive oil, FS17 Favolosa, Coratina, hydroxytyrosol, oleacein, oleuropein aglycone, ligstroside aglycone, cell metabolism, enzymatic activities

## Abstract

The effects of the phenolic compounds of extra virgin olive oil (EVOO) on AGS cells have never been studied so far, which is the aim of this study. The profiles of the main phenolic components in EVOOs, mainly secoiridoid compounds derived from the transformation of oleuropein during the olive milling process, were evaluated and compared. Oils of different origins were evaluated aiming at verifying whether chemical differences in the phenolic composition of the dry extracts played a role in the metabolism and in maintaining the cellular redox state of AGS cells. The following key enzymes of some metabolic pathways were studied: lactate dehydrogenase, enolase, pyruvate kinase, glucose 6-phosphate dehydrogenase, citrate synthase, 3-Hydroxyacyl-CoA dehydrogenase and hexokinase. As confirmed through PCA analysis, pretreatments with the dry extracts of EVOOs at different concentrations appeared to be able to counteract the enzymatic activity alterations due to oxidative stress induced by H_2_O_2_ 1 mM and 2 mM. The studied phytocomplexes showed the ability to protect AGS cells from oxidative damage and the secoiridoid derivatives from both oleuropein and ligstroside contributed to the observed effects. The results suggested that EVOOs with medium to high concentrations of phenols can exert this protection.

## 1. Introduction

New lifestyle habits are linked to a growing demand for foods able to maintain well-being, and extra virgin olive oil (EVOO) is certainly included within the healthy foods [1,2,3,4,5]. Among the commercial edible oils, only the EVOOs contain appreciable amounts of phenolic compounds. In particular, fresh EVOOs are rich in secoiridoid molecules derived from oleuropein and ligstroside. These two precursors are the glycosides of olive fruit that, by the action of native β-glucosidases, are rapidly transformed into several de-glycosylated molecules during the milling process [6,7]. Oleacein and oleocanthal are among the main secoiridoids with a di-aldehydic structure, which also determine some peculiar sensory properties of EVOO, such as bitterness and pungency, respectively [8,9]. Concerning the nutraceutical aspects of EVOO, since 2011, European Food Safety Authority (EFSA) permitted some health claims related to the phenolic content, in particular, to the amount of hydroxytyrosol (OH-tyr) in the oil [5,10]. The phenolic amount of the EVOO can be an instrument for better defining the nutraceutical value of this food, even if this parameter is not applied for the classification of the different categories of the virgin olive oils [11].

The scientific results on the protective effects in humans exerted by the phenolic compounds of EVOO are numerous and have mainly involved cardiovascular protection [5], but also the reduction in blood sugar in type II diabetes [12], or a protection in mild Alzheimer’s disease [13,14]. The beneficial effects of EVOO phenols can be mediated by several biochemical pathways, which are partially elucidated by in vitro tests, for example, on CaCo2 cells [15]. Signaling mechanisms can act independently or synergistically; however, most of these works have highlighted the antioxidant properties of these molecules.

Cells and, therefore, organisms in normal conditions maintain a redox homeostasis. When there is an imbalance caused by an increase in reactive oxygen species (ROS) production, a situation of oxidative stress occurs [16]. A prolonged exposure of cells to oxidative stress contributes to the development of various diseases also in the gastrointestinal tract. At an intestinal level of oxidative stress, it is involved in serious pathologies, such as inflammatory bowel diseases (IBD), enteric infections and colorectal cancer [17,18,19].

Oxidative stress is implicated also in severe gastric disease, gastritis, peptic ulcer, gastric adenocarcinoma [20] and diseases based on microbial infections. *H. pylori* infection is responsible for an inflammatory state with high and long-term oxidative stress that represents one of the first steps of the Correa cascade that leads to gastric adenocarcinoma [21,22,23]. Therefore, there is a serious need to develop preventive and therapeutic approaches that reduce oxidative stress, and, for this purpose, bioactive compounds present or to be added in the diet can be a great resource. These compounds can be useful to prevent and mitigate oxidative stress injury [17].

To the authors’ knowledge, no study has been conducted on the effects of phenolic compounds extracted from EVOO on the metabolism of AGS cells. A work by Kountouri et al. [24] investigated the effects of phenolic extracts from olive fruits on AGS cells, but the extracts of phenolic compounds from the fruit have a composition different from those of EVOO. The data from the work of Peri and colleagues highlighted the ability of a phenolic extract from EVOO to affect the viability and the cloning efficiency of AGS wild-type cells [25]. Analogously, the cytotoxic and antiproliferative activity of six secoiridoids extracted from EVOOs were evaluated and compared on several cancer cell lines, including AGS cells [26]. Recently, a similar approach was applied to study the phenolic phytocomplex of a by-product of virgin olive oil production named pâté and characterized by a different phenolic profile [27]. In this context, the present research aimed to evaluate how phenolic extracts from monocultivar EVOOs with a different phenolic content affect proliferation, migration and redox status of AGS cells. This gastric cell line derived from an adenocarcinoma is also used as a model of gastric mucosa and maintains the ability to respond to oxidative stress of nontumor cells [28,29,30]. Afterwards, the possibility that the extracts can be used as a countermeasure to enzymatic activity alterations due to an oxidative stress induced by H_2_O_2_ was investigated; the activities of seven key enzymes of some of the main cellular metabolic pathways (glucose phosphorylation and its entrapment in the cell, glycolysis, cytoplasmatic NADH re-oxidation, pentose phosphate shunt, Krebs cycle and beta oxidation) were assayed in cells pretreated with two different concentrations of extracts and then subjected to H_2_O_2_-induced oxidative stress. The choice of oils of different origins was aimed at verifying whether chemical differences in total phenolic content and hydroxytyrosol content of the corresponding dry extracts play a role in the metabolism of these cells.

## 2. Materials and Methods

### 2.1. Phenolic Extracts from Extra Virgin Olive Oils (EVOOs)

Five monovarietal EVOO samples (one of each of the following cultivars: Frantoio, Leccino, Favolosa, Coratina and Tonda Iblea, hereafter also indicated as oil 1, oil 2, oil 3, oil 4 and oil 5, respectively) were purchased from different Italian producers in the 2020 olive oil campaign. Phenolic extracts were obtained from each sample starting from 50 g of oil, which were extracted with 150 mL of MeOH:H_2_O 80:20 *v*/*v* solution. The mixture was shaken for 1 min and then for 15 min in an ultrasound bath at room temperature; then, it was centrifuged at 5000× *g* rpm for 25 min at 15 °C; the supernatant was filtered, defatted twice with 150 mL of hexane and evaporated under vacuum at 35 °C. The residue was recovered with 6 mL EtOH, transferred into a small flask and evaporated again; the dry weight of the extracts was measured. The final extract was recovered with 10 mL EtOH, split into 10 vials and dried again under nitrogen flow. This step was applied to guarantee the chemical stability over time of the extracts before the following biological tests. The mean weights of extract in each vial, corresponding to 5 mL of oil, were as follows: Frantoio, 4.31 mg; Leccino, 3.09 mg; Favolosa, 4.59 mg; Coratina, 7.63 mg; Tonda Iblea, 1.97 mg.

The obtained extracts were also subjected to the acid hydrolysis method previously validated [10]; briefly, 300 μL of phenolic extract was added to a 2 mL vial together with 300 μL of 1 M H_2_SO_4_; the obtained solution was kept at 80 °C for 2 h in an oven; after that, 400 μL of water was added and the obtained hydrolyzed extract was subjected to chromatographic analysis.

### 2.2. Analyses by HPLC-DAD-MS of the Extracts

The chromatographic analysis of the phenolic extracts obtained as described above was carried out using two different methods: the IOC official method [31] and a method developed by our research group [32]. Briefly, the chromatographic conditions applied for the IOC method were the following: an HP1100 liquid chromatograph coupled with a DAD was used, and separation of phenols was carried out by using a C18 SphereClone ODS column (5 μm, 250 × 4.6 mm id; Phenomenex, Bologna, Italy); acetonitrile, methanol and water (pH 2.0 with H_3_PO_4_) were used as the elution solvents, following the elution gradient and the flow rate reported in the official method. Quantification was performed by mean of the internal standard method, using syringic acid as the IntSTD and tyrosol (Tyr) as reference compound; therefore, results were expressed as mg_tyr_/kg_oil_.

The second method was applied using an HPLC instrumentation (1260 Infinity II LC System) provided with both Diode Array Detector (DAD) and Mass Spectrometry Detector (InfinityLab LC/MSD) with an APIelectrospray interface (all from Agilent, Santa Clara, CA, USA). In this case, a new generation Poroshell 120, EC-C18 column (150 mm × 3.0 mm id, 2.7 μm particle size) from Agilent Technologies was employed to improve the peaks’ resolution. The column temperature was kept at 26 °C. Acetonitrile (A) and water (pH 3.2 HCOOH) were used as the elution solvents, applying a flow rate of 0.4 mL min^−1^ and a multistep linear gradient as follows: solvent A from 5% at 0.1 min to 40% at 40 min, to 88% in 5 min and to 98% in 10 min, staying at 98% for 3 min, for a total analysis time of 58 min. Chromatograms were recorded at 280 nm because this wavelength corresponds to the typical absorption maximum of most phenolic molecules of EVOO. Conditions of the MS detector were as follows: it acquired in negative ion mode, applying a capillary voltage of 3500 V, fragmentation energy of 150 V, a flow rate of nitrogen of 10.5 L/min, a gas temperature of 350 °C and a pressure of the nebulizer of 35 psi (241 KPa).

Finally, the HPLC-DAD analysis of the hydrolyzed extracts was performed using an HP1100 liquid chromatograph coupled with a DAD (Agilent Technologies, Santa Clara, CA, USA). Phenolic compounds were separated using a 150 × 3 mm (5 μm) Gemini RP18 column (Phenomenex, Torrance, CA, USA). The elution solvents water (pH 3.2, formic acid, solvent A) and acetonitrile (solvent B) were used, applying a flow rate of 0.4 mL min^−1^. A multistep linear gradient was applied as follows: solvent A started from 95% and diminished to 70% in 5 min, to 50% in 5 min and to 2% in 5 min, with a final plateau of 5 min, for a total analysis time of 22 min. Chromatograms were recorded at 280 nm and results were expressed as mg_tyr_/kg_oil_ for tyrosol, mg_OHtyr_/kg_oil_ for hydroxytyrosol (for the latter molecules, after application of the correction factor proposed by [33], useful for considering the 35% overestimation of OH-Tyr when the calibration curve of tyrosol is used).

### 2.3. AGS Cell Line

AGS cells (ATCC CRL-1739) are derived from a gastric adenocarcinoma but, also, are commonly used as a model of gastric mucosal cells. The cells were purchased from Merck (Darmstadt, Germany) [34] and were grown in F-12 Kaighn’s modification medium (Hyclone, GE Healthcare Lifesciences, Marlborough, MA, USA) with 10% fetal bovine serum (FBS, Biowest, Nuaillé, France) at 37 °C in a 5% CO_2_ humidified atmosphere. A total of 1 × 10^6^ cells were seeded in 10 cm Petri dishes and propagated every 2 days by treatment for 5 min with a 0.25% trypsin/EDTA solution (MERCK, Darmstadt, Germany). Cultures were periodically tested for *Mycoplasma* spp. contamination.

### 2.4. Assessment of Cell Viability by MTT Test

The effects of the oils on AGS cell viability were assessed by MTT test; 12,000 cells/well were seeded in a 96-multiwell plate and, after 48 h, were incubated for 2, 24 and 48 h with various concentrations (5, 10, 20, 50, 100 and 200 µg/mL) of EVOO extracts reconstituted with dimethyl sulfoxide (DMSO). Cell controls were incubated with DMSO only.

After incubation, cell viability was measured by incubating with 1 mM thiazolyl blue tetrazolium bromide (MERCK, Darmstadt, Germany) in culture medium for 40 min in the dark. Then, the medium was removed and DMSO was added to dissolve formazan crystals. The absorbance signal at 570 nm was read on the multiplate reader (Infinite M200PRO, Tecan, Mannedorf, Switzerland) and, to obtain normalized absorbance values, the background absorbance at 630 nm was subtracted from signal absorbance.

### 2.5. Cell Distribution in the Cell Cycle Phases by Cytofluorimetric Analysis

For the analysis of the distribution of the AGS cells in the different phases of cell cycle after incubation with oils at the concentrations of 10 and 20 µg/mL for 2, 24 and 48 h, 250,000 cells were seeded in 6 cm Petri dishes and, after 48 h, incubated with the extracts. At the end of incubation, the cells were collected by trypsinization and were stained with propidium iodide according to the method of Vindeløv [35]. The analysis of the samples was carried out using an FACScan flow cytometer (Becton Dickinson, Milan, Italy). Quantification of cells in the different cycle phases was performed by ModFit LT software, version 3.0 (Verity Software House Inc., Topsham, ME, USA).

### 2.6. Scratch Assay

A total of 2.5 × 10^5^ AGS cells were seeded in 6 cm Petri dishes and cultured until ∼80% of confluence. Then, the monolayers were gently scratched with a 200 μL pipette tip. Culture medium and detached cells were removed and monolayers were washed twice with phosphate buffer solution (PBS). Fresh complete medium with 10 μg/mL of oil extracts was added to cells (in control samples, fresh complete medium without oils was added). Samples were then observed at the microscope as detailed in the following subsection; this moment is considered 0 delay time of the scratch closure to which we refer in the following.

Experiments were performed, realizing twin scratch assays with the role of control and treat. Each control–treat experiment was performed in triplicate.

Scratch assay imaging was performed with an inverted microscope in phase contrast configuration (Leica DM IL, Leica Microsystems GmbH, Wetzlar, Germany) equipped with a 5× objective and a Canon CCD camera (Canon Power shot S40, 2272 × 1074, ∼23 × 20 μm pixels, Canon Inc., Tokyo, Japan), obtaining a field of view of ∼2.5 × 2.5 mm necessary to observe the entire scratch width (∼0.6–1.4 mm). Images of the scratch were acquired approximately once an hour (except for the unavoidable night-time gap) from the starting time to ∼25–40 h, thus guaranteeing 10–12 images. 

Dedicated image analysis routines written in python language (Python Software Foundation, version 3.8, available at https://www.python.org/) allowed the scratch width to be measured for each image. Then, for each sample and delay time, mean scratch width and standard deviation on the triplicate was calculated. Finally, the scratch width vs. delay time curve for each sample was constructed.

This curve was then modelled by means of least squares minimization with a linear model calculating the scratch closure velocity and closure time (i.e., the time at which the scratch is completely closed according to the linear model).

For any detail regarding image analysis and scratch closure modelling, refer to [36].

### 2.7. Assessment of Intracellular ROS Production

Intracellular ROS production was quantified by assay with 2′,7′-dichlorofluorescein diacetate (DCF-DA, Merck, Darmstadt, Germany) as described by Bass et al. [37]. Cells were seeded in a 96 multiwell (18,000 cells/well) and, after 24 h, were incubated with oil extracts (10 and 20 µg/mL) and 25 μM DCF-DA (dissolved in culture medium) for a day. Then, the cells were treated with H_2_O_2_, 1 or 2 mM, for 1 h. These H_2_O_2_ concentrations were chosen because they determine a detectable ROS production in AGS cells. At the end of treatment with H_2_O_2_, samples were washed twice with PBS immediately; the relative levels of fluorescence emission were quantified in the multiplate reader (excitation: 485 nm, emission: 535 nm).

### 2.8. Enzymatic Assays

For the determination of the activities of the assayed enzymes, representative of the principal metabolic pathways, 4 × 10^5^ cells were seeded in 10 cm Petri dishes. After one day, 24 h incubation with 10 and 20 µg/mL of oil 3 and oil 4 started. Then, the cells were treated with H_2_O_2_ 1 or 2 mM for 1 h and, at the end of the treatment, the cells were collected by trypsinization.

Cells were quickly rinsed in ice-cold PBS and frozen. At the time of use, after thawing the samples at room temperature and 30 min of incubation on ice, cells were lysed by sonication (three short bursts) at 4 °C in 50 mM Tris, pH 7.4, containing 5 mM dithiothreitol and Sigma protease inhibitors mix (1/100, *v*/*v*) and centrifuged at 12,000× *g* rpm in a microcentrifuge at 4 °C for 30 min.

Total protein content [38] and enzymatic activities of the assayed enzymes were quantified in supernatants.

The determinations of the enzymatic activities of hexokinase (HK, EC 2.7.1.1), enolase (ENO, EC 4.2.1.11), pyruvate kinase (PK, EC 2.7.1.40), lactate dehydrogenase (LDH, EC 1.1.1.27), glucose-6-P-dehydrogenase (G6PDH, EC 1.1.1.49), citrate synthase (CS, EC 4.1.3.7) and 3-Hydroxyacyl-CoA dehydrogenase (HACoADH, EC 1.1.1.35) were performed at 37 °C, according to Bergmeyer [39], with slight modifications, continuously following NADH or NADPH appearance/disappearance at 340 nm, using a UV-2100 spectrophotometer (Shimadzu, Columbia, MD, USA).

All the enzymatic reactions were started by adding the substrate.

One unit of activity is defined as the quantity of enzyme which transforms 1 μmol of substrate in 1 min. The value of 6.22 mM^−1^ cm^–1^ is considered the NADH (or NADPH) molar extinction coefficient.

All the reagents used for enzymatic assays were purchased from Merck (Darmstadt, Germany).

### 2.9. Principal Component Analysis (PCA) of Enzymatic Activities

PCA analysis [40] was performed to discuss the influence of the investigated treatments on the global profile of the 7 measured enzymatic activities. In each of 3 experiments, the following samples were prepared in triplicate: control, H_2_O_2_ 1 mM, H_2_O_2_ 2 mM, extract of oil 3 at 10 µg/mL, extract of oil 3 at 20 µg/mL, extract of oil 4 at 10 µg/mL, extract of oil 4 at 20 µg/mL, H_2_O_2_ 1 mM with 10 µg/mL extract of oil 3, H_2_O_2_ 2 mM with 20 µg/mL extract of oil 3, H_2_O_2_ 1 mM with 10 µg/mL extract of oil 4 and H_2_O_2_ 2 mM with 20 µg/mL extract of oil 4. Each PCA data point represents the average of each of the above-described samples on one experiment. The principal components were calculated, and the results were plotted in the form of biplots with the two principal components accounting for the highest fraction of the original data variance. PCA analysis was performed separately for the two concentrations of each oil 3 and oil 4 and, being the decomposition technique based on the data variance, inserting in a unique dataset different oil concentrations or different oils could increase the data variance and lead to an incorrect interpretation of the results. Therefore, PCA was performed independently for the datasets relative to the dose of 10 µg/mL extract of oil 3, 20 µg/mL extract of oil 3, 10 µg/mL extract of oil 4 and 20 µg/mL extract of oil 4.

### 2.10. Statistical Analysis

All the experiments were carried out in triplicate. The differences between treated and control samples observed in biological activity assessment assays, such as MTT test, flow cytometry, ROS production assay and enzymatic activity, were analyzed by Student’s *t*-test (significance level of 0.05). Moreover, to evaluate the statistical significance of the trend over time of MTT test and enzymatic activities assay, a two-way analysis of variance was carried out (ANOVA test with a significance level on the interaction parameter of 0.05). ANOVA and Student’s *t*-test were performed by GraphPad Prism version 5.03 (GraphPad Software, San Diego, CA, USA, www.graphpad.com).

## 3. Results and Discussion

### 3.1. Characterization of the Phenolic Profiles

For the quantification of the phenolic extracts of the EVOOs, each sample was analyzed before and after acid hydrolysis [10]. The analysis of the extracts before hydrolysis made it possible to obtain the distribution of the different phenols in the samples, in particular secoiridoids derivatives, which are the most abundant phenols in fresh extra virgin olive oils (EVOOs), and simple phenols. The analysis of the hydrolyzed extracts, instead, made it possible to quantify the total content of tyrosol and hydroxytyrosol in the free and bound forms, thus allowing for a better estimation of the total content of ligstroside and oleuropein derivatives, respectively. A summary of the results expressed as mg/kg of oil are shown in Table 1. Since the biological assays were carried out using the dry phenolic extract (DE), the phenolic amount was calculated in these samples (Table 2).

The richest oil in terms of total phenols before acid hydrolysis is from the Coratina cultivar (from Apulia), followed by that of the Favolosa (from Apulia) and Frantoio (from Tuscany) cultivars, while the oil from the Tonda Iblea cultivar (of Sicilian origin) shows the lowest phenolic content. As for the content after acid hydrolysis, the oils from the Coratina and Tonda Iblea cultivars were richer in tyrosol than in hydroxytyrosol, suggesting a higher content in ligstroside derivatives than in oleuropein derivatives. In the oils of the other three cultivars, the hydroxytyrosol was predominant, indicating a higher quantity of oleuropein derivatives. The data in Table 1 highlight that the selected EVOOs showed a variability in their phenolic profiles and, therefore, they can be suitable to represent the biodiversity of the phenolic pattern of EVOOs.

The data are expressed as phenol content on DE (Table 2) evidence differences in the weight of the dry extract obtained from 100 g of oil, with values ranging from 69.8 mg (Tonda Iblea) to 162.2 mg (Coratina). Concerning the phenolic concentration, differently from the data expressed on the oil weight (Table 1), the results for the five samples were more similar among each other, with a maximum for Favolosa of 507 mg/g DE and a minimum from Tonda Iblea of 355.8 mg/g DE. The ratio between the total content of hydroxytyrosol and tyrosol obtained after hydrolysis allowed two groups of samples to be defined: a group constituted by Frantoio and Leccino e Favolosa, with values ranging from 1.14 to 1.15, and a second group constituted by Coratina and Tonda Iblea, with lower values of 0.72 and 0.52, respectively. According to data in Table 2, the dry extracts of Favolosa and Coratina cultivars were selected to develop further biological assays because they are characterized by a high phenolic content but a different ratio of OH-Tyr/Tyr.

Moreover, in the last decade, the Favolosa cultivar has become very important in the context of olive growing, since it shows a particular tolerance towards infection by *Xilella fastidiosa* and is, therefore, used as the main cultivar for the reforestation of Salent after the pandemic occurred from 2013 in the province of Lecce (Apulia, Italy).

**Figure 1:**
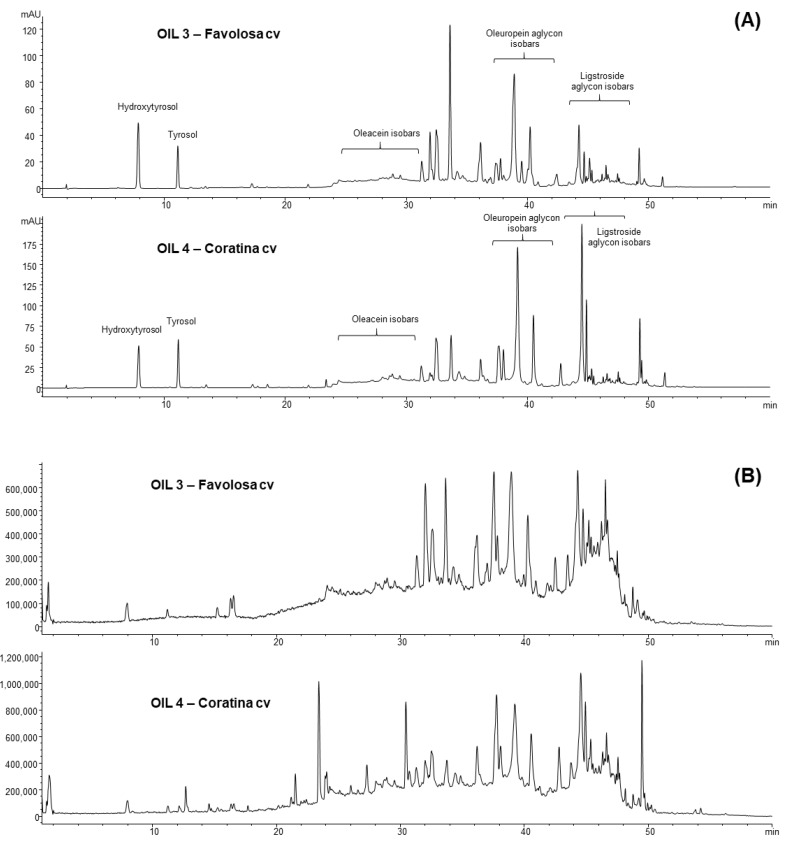
(**A**) Different phenolic profiles at 280 nm of the two EVOOs Favolosa and Coratina; the analyses were performed according to the method of Bartolomei et al. [32]; (**B**) comparison of the TIC profiles obtained applying fragmentor 200 V as for both the extracts of Coratina and Favolosa.

**Figure 2:**
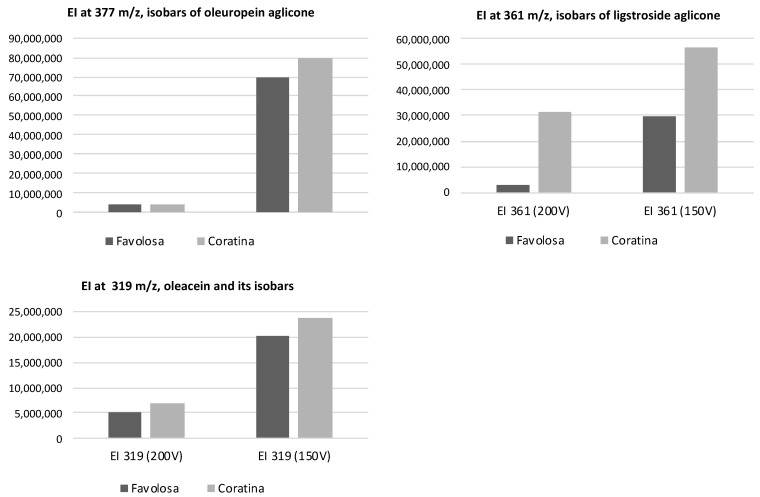
Comparison of the area values (counts from MS) for the dried phenolic extracts of Favolosa and Coratina oils evaluated at *m*/*z* 377 (oleuropein aglycone and its isobars), at *m*/*z* 361 (ligstroside aglycone and its isobars) and at *m*/*z* 319 (oleacein and its isobars). The area values were obtained in negative ionization mode at two different fragments of 150 V and 200 V.

To investigate the differences in the chemical composition of these two extracts, particularly for the secoiridoid derivatives of oleuropein and ligstroside, a further HPLC-DAD-MS analysis was carried out according to the method previously described [29]. The profiles at 280 nm and the corresponding TIC for the two samples are shown in Figure 1. The data in Figure 2 compare the area values of the extract ions at diagnostic *m*/*z* values typically used to identify the oleuropein aglycone and its isobars (*m*/*z* 377), the isobars of ligstroside aglycone (at *m*/*z* 361) and the oleacein (*m*/*z* 319), a further derivative of oleuropein aglycone usually abundant in fresh EVOOs. The isobaric forms of oleuropein and ligstroside aglycones are usually constituted by mono and di-aldehydic derivatives [6]. As clearly shown, the main differences were related to the ligstroside derivatives, largely more concentrated in the oil from the Coratina cultivar. As for the oleuropein derivatives, the differences were less relevant. The data were in agreement with the lower value of Coratina with respect to Favolosa for the ratio between total yydroxytyrosol/tyrosol determined after acidic hydrolysis (Table 2).

### 3.2. Cell Viability by MTT Test and Distribution in the Cell Cycle Phases by Cytofluorimetric Analysis

MTT test was applied to all the phenolic extracts to define a range of concentration that did not modify the cell viability. Preliminary results carried out with the phenolic extracts of the five EVOOs (at different incubation times) suggested that concentrations below 50–25 µg/mL showed results similar to that of the control. In the next step, two phenolic extracts rich in phenols but with different chemical profiles (Figure 1: oil 3 (Favolosa cv) and oil 4 (Coratina cv)) were selected.

**Figure 3:**
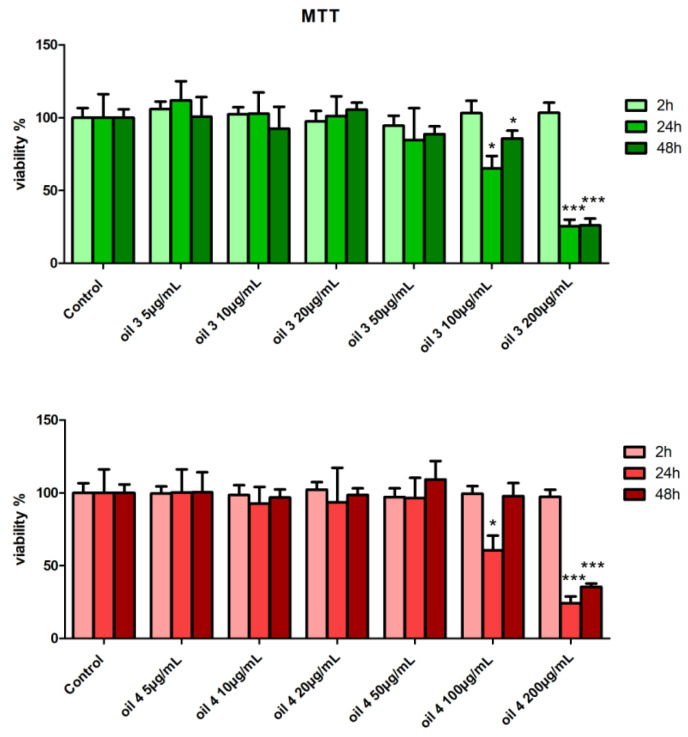
Cell viability after treatment with the extracts of oil 3 (top panel) and oil 4 (bottom panel) at the concentration of 10 and 20 µg/mL for 2, 24 and 48 h by MTT assay. Samples significantly different from control at the same time are marked with * (Student *t*-test *p* < 0.05) or *** (*p* < 0.001).

More specifically, the results of MTT test after incubation with the extracts of oil 3 and oil 4 showed no significant alteration in cell viability with respect to the control for concentrations up to 50 µg/mL of both oils at the three incubation times (2, 24 and 48 h) (Figure 3). For the concentration of 100 µg/mL cell viability shows a significant decrease in the samples treated with oil 3 for 24 and 48 h and in the sample treated with oil 4 for 24 h (*p* < 0.05). At the concentration of 200 µg/mL and incubation times of 24 and 48 h, cell viability decreased drastically for both the oils compared to the control (*p* < 0.001). These observations suggested that the extracts of both oils are not cytotoxic in the concentration range between 5 and 50 µg/mL.

Therefore, two not cytotoxic concentrations, 10 and 20 µg/mL, were chosen for the subsequent analyses.

**Figure 4:**
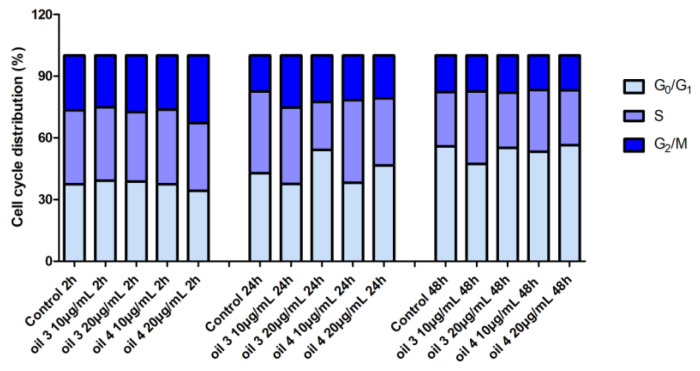
Percentage distribution in the different phases of cell cycle of samples treated with 10 and 20 µg/mL of the extract of oil 3 and oil 4 for 2 h, 24 h and 48 h.

The analysis of the cell cycle phase distribution by flow cytometry in samples treated with oil 3 and oil 4 extracts at the concentrations of 10 and 20 µg/mL for 2, 24 and 48 h did not show statistically significant difference with respect to the control sample at the same times (Figure 4).

These results are in agreement with what was observed by MTT test and indicates no remarkable alterations in proliferative activity in samples treated with 10 µg/mL and 20 µg/mL of both oil 3 and oil 4.

### 3.3. Scratch Assay Results

**Figure 5:**
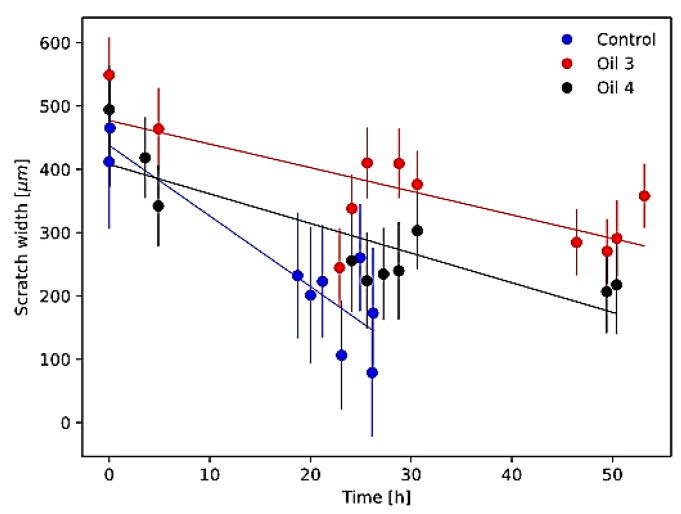
Scratch closure curves (colored circles, with standard deviation error bars) and relative best-fit linear models (continuous lines). Blue circles refer to the control cell sample, red circles refer to the treatment with 10 µg/mL of the extract of oil 3 and black circles to the treatment with 10 µg/mL of the extract of oil 4.

In Figure 5, the measured scratch closure curves for the investigated samples are shown together with the best-fit linear models. In Table 3, the measured closure velocity and closure time for the investigated samples are reported.

Scratch assay closure modelling results showed a statistically significant reduction in scratch closure velocity with respect to control for both oil 3 and oil 4 (and, therefore, an increase in the scratch closure time). This indicates that both oil 3 and oil 4 at the concentration of 10 µg/mL reduced the cellular migration and proliferation with respect to the control.

On the other hand, the cell viability and cycle phase distribution assays showed that the concentration of 10 µg/mL of both oil 3 and 4 does not change the proliferation of AGS cells with respect to the control. This suggests that these 10 µg/mL treatments could reduce the cell migration while not changing their proliferation. In the literature, instead, various findings are present: cell lines (tumoral and not) treated with natural compounds with antioxidant properties often show a migration inhibition coupled with cytotoxicity but, also, migration inhibition coupled with low cytotoxicity or even migration stimulation [41,42,43,44,45,46,47]. These results deserve more in-depth investigations that will be performed in the future, starting with enlarging the investigated extract concentration range.

### 3.4. Intracellular ROS Production Assessment

**Figure 6:**
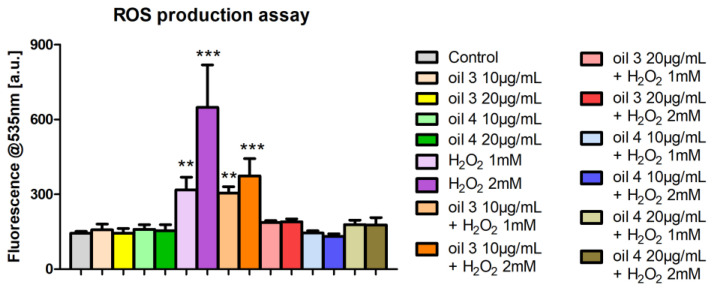
ROS production assessed by DCF-DA test in cells treated with the extracts of oil 3 and oil 4 at the concentrations of 10 and 20 µg/mL for 24 h and then incubated with two different concentrations of H_2_O_2_, 1 mM or 2 mM, for 1 h. ** and *** indicate a statistically significant difference compared to the control sample (Student’s *t*-test ** *p* < 0.01, *** *p* < 0.001).

The assessment of ROS production by DCF-DA assay indicated that the treatments with the two EVOO extracts at the chosen concentrations (10 and 20 µg/mL) produced ROS levels comparable to the control ones. The treatment with oil 3 at 10 µg/mL and both H_2_O_2_ 1 mM or 2 mM did not show a reduction in ROS production with respect to cells incubated with only H_2_O_2_ 1 mM or 2 mM, while the same oil extract at the concentration of 20 µg/mL determined a decrease in the ROS levels with respect to the samples treated with only the two H_2_O_2_ concentrations. The extract of oil 4 at 10 and 20 µg/mL showed ROS levels analogous to control sample for both 1 mM and 2 mM H_2_O_2_ concentrations (Figure 6).

These results indicate that hydrogen-peroxide-induced stress is efficiently counteracted by oil 3 extract at 20 µg/mL and by oil 4 extract at 10 and 20 µg/mL and these observations clearly demonstrate the antioxidant capacity of the tested EVOO extracts.

### 3.5. Enzymatic Assays and PCA

Some preliminary enzymatic assay tests were performed with the phenolic extracts of the five EVOOs, as already conducted for the MTT test. The results highlighted a clear increase in the activity levels of LDH by the five phytocomplexes, indicating the ability to induce a stimulating–activating effect on cellular metabolism. To better understand and confirm these preliminary data, further tests with seven key enzymes were carried out, working with the phenolic extract of oil 3 and oil 4. In particular, the specific activities of the following seven key enzymes of important metabolic pathways were determined in AGS cells: LDH (cytoplasmatic NADH reoxidation), G6PDH (pentose phosphate pathway), ENO and PK (glycolysis), CS (Krebs cycle), HA-CoADH (beta oxidation) and HK (glucose phosphorylation)

The enzymatic assays with oil 3 and oil 4 were performed, pretreating (or not) with the two phytocomplexes at the concentrations of 10 and 20 µg/mL for 24 h and subsequently treating (or not) with H_2_O_2_ 1 mM or 2 mM to induce oxidative stress. The aim was to investigate their metabolic effect but also their involvement in maintaining the redox state of the cell, given the close relationships that exist between metabolism and oxidative stress [48,49].

**Figure 7:**
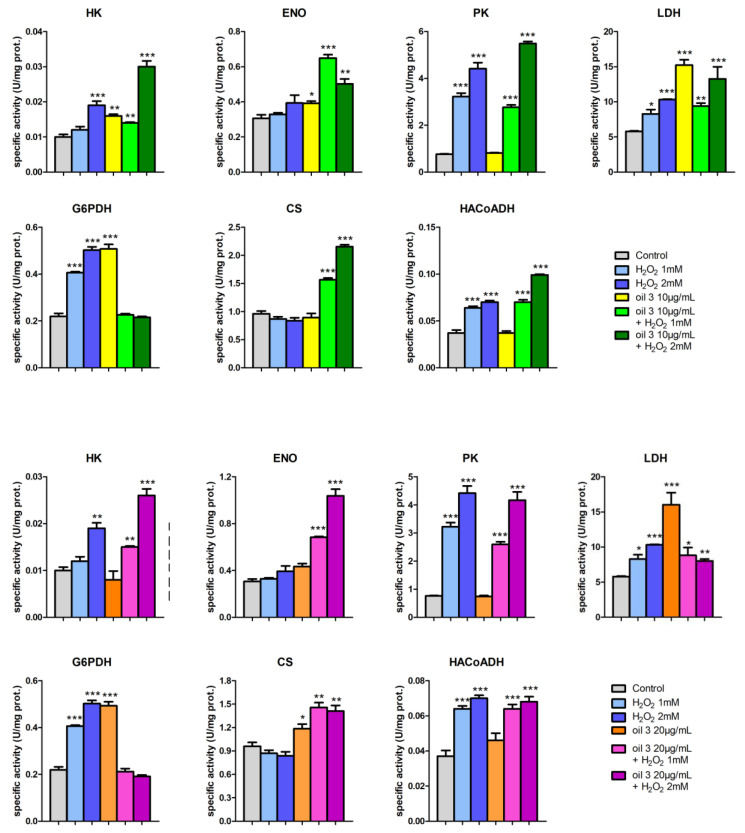
Specific activities of (from left to right and from top to bottom of each panel) HK, ENO, PK, LDH, G6PDH, CS and HACoADH and for the two investigated concentrations of the extract of oil 3: top panel with 10 µg/mL and bottom panel with 20 µg/mL. For each enzyme, the activities were measured for control cells, cells treated with H_2_O_2_ 1 mM and 2 mM, 10 µg/mL or 20 µg/mL of the extract of oil 3 and cells treated with both the two concentrations of H_2_O_2_ and of oil 3, as specified in the legend. Error bars represent the standard deviation on the three replicates of the assay. Samples significantly different from control are marked with * (Student *t*-test *p* < 0.05), ** (*p* < 0.01) or *** (*p* < 0.001).

**Figure 8:**
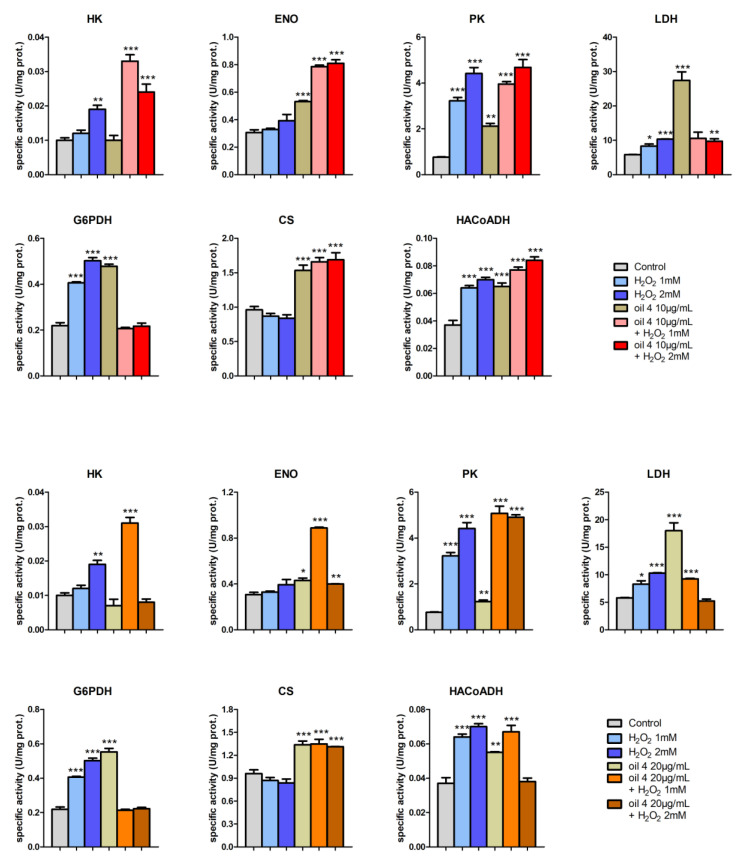
Specific activities of (from left to right and from top to bottom of each panel) HK, ENO, PK, LDH, G6PDH, CS and HACoADH and for the two investigated concentrations of the extract of oil 4: top panel with 10 µg/mL and bottom panel with 20 µg/mL. For each enzyme, the activities were measured for control cells, cells treated with H_2_O_2_ 1 mM and 2 mM, 10 µg/mL or 20 µg/mL of the extract of oil 4 and cells treated with both the two concentrations of H_2_O_2_ and of oil 4, as specified in the legend. Error bars represent the standard deviation on the three replicates of the assay. Samples significantly different from control are marked with * (Student *t*-test *p* < 0.05), ** (*p* < 0.01) or *** (*p* < 0.001).

Figure 7 and Figure 8 show the specific activity values of the seven enzymes evaluated for different treatments carried out on cultured AGS cells. Each bar represents the average and standard deviation of the measurements on three replicate assays. 

Very interesting is the framework of LDH, whose activity increased significantly following stimulation with H_2_O_2_. In the literature there are many works in which lactate is considered not only as the central molecule of all metabolism, but also closely involved in maintaining the redox state of the cell with a signaling role [50]. Through the catalytic action of LDHA (reduction of pyruvate to lactate by oxidation of NADH to NAD^+^) and LDHB (oxidation of lactate to pyruvate by reduction of NAD^+^ to NADH), the NADH/NAD^+^ ratio is checked (regulated) and then the cytoplasmic redox state of the cell. In addition, the presence of a specific lactate transporter in the inner membrane of the mitochondria allows lactate to be translocated from the cytoplasm to the mitochondrial matrix, where, thanks to LDHB, it is converted to pyruvate with the reduction of NADH, conditioning the mitochondrial redox state [51].

LDHA is also considered a sensor for ROS and an increment of their concentration upregulates the transcription of LDHA through HIF1a [52,53].

The same increase in LDH activity, but much more marked, was observed even after treatment with extracts from oil 3 and oil 4 (Figure 7 and Figure 8).

In Figure 6, it is evident that the two phytocomplexes, unlike H_2_O_2_, did not induce production of ROS and, therefore, we can assume that the same effect (increase in LDH activity) was induced by different causes. It is known that inhibition of LDH increases levels of oxidative stress and cell death in many cell models, while its activation produces an opposite effect [54].

In this context, therefore, oil 3 and oil 4 extracts can be considered as able to counteract oxidative stress by activating LDH. In fact, if oxidative stress with H_2_O_2_ was induced after pretreatment with the phytocomplex, we did not observe a summation of the two effects but, rather, the pretreatment tended to bring the activity of the LDH towards the control values; this was especially evident for oil 4 at the maximum tested concentration. On the other hand, the analysis of the two phytocomplexes (Table 2) highlighted that they were different in their composition, in particular, for the different tyrosol and hydroxytyrosol ratio, although the total phenolic amount in the dry extract was similar (507 mg/g DE and 460.2 mg/g DE for oil 3 and 4, respectively). The extract from oil 3 was richer in hydroxytyrosol and, therefore, in oleuropein derivatives, while the extract from oil 4 showed opposing characteristics, with a greater amount of ligstroside derivatives resulting from the higher concentration of total tyrosol. Oil 4 shows a much more marked effect than oil 3 to equal treatment.

Very interesting also is the framework of G6PDH (Figure 7 and Figure 8), a key enzyme of the pentose phosphate pathway (PPP) involved in the production of NADPH and, therefore, in the maintenance of glutathione in its reduced state with the function of scavenger against ROS. An oxidative state induced by H_2_O_2_ caused an increase in activity levels of G6PDH, with a mechanism mediated by ROS, as widely described in the literature [49] and, therefore, a shift in glucose-6 phosphate (G6P) from glycolysis to PPP.

Oil 3 and oil 4 had an effect with a similar trend to that of H_2_O_2_ but, also in this case, with different causes, since they did not induce production of ROS (Figure 6). In fact, the action of H_2_O_2_ was affected by the pretreatment with the two phytocomplexes that kept the activity of G6PDH at the level of control, as if they could predispose the cell towards a set-up (availability of NADPH) able to counteract an increase in ROS.

The framework of CS activity levels shows that oil 3 and oil 4 had a protective and even stimulating effect on the enzyme (therefore, on the functionality of the Krebs cycle and mitochondria) against oxidative stress induced by H_2_O_2_.

As for ENO (Figure 7 and Figure 8), the effect of oil 3 and oil 4 on the activity of the enzyme had a similar trend to that of CS.

The activity levels of PK (Figure 7 and Figure 8) contrasted with those of the literature, in which it is reported that oxidative stress has a depressing effect on the activity of the enzyme. This effect is directed towards the M2 isoform of the PK (PKM2), which is oxidized and then inactivated at the cys358 by ROS [55] and AGS cells present PKM2.

Our data show an increase in PK activity levels in the case of treatment with H_2_O_2_, both in the absence and in the presence of pretreatment with oil 3 and oil 4. Of the two phytocomplexes, only oil 4 seemed to have a slight activating effect on the PK.

This behavior could be simply explained with the conditions of cell lysis for the preparation of the extracts used for the enzymatic assays that do not preserve the reduced state of cys358 and, therefore, the sensitivity of PK activity to oxidative stress with H_2_O_2_.

Treatment with the highest concentration of H_2_O_2_ led to an increase in the level of activity of HK. It is known that ROS can promote the entry of glucose into the cell, with a consequent increase in the HK activity [56]. Pretreatment with oil 3 and oil 4 seemed to amplify the effect of H_2_O_2_.

As for the activity levels of HACoADH (Figure 7 and Figure 8), oil 3 had no effect on the enzyme, while oil 4 increased the activity of the enzyme in a similar way to H_2_O_2_. Pretreatment with the two phytocomplexes did not preserve from the effect of H_2_O_2_.

The fact that the activity levels of the enzymes tested continued to remain higher than the controls, even in the case of cells treated with H_2_O_2_ after pretreatment with the two EVOO extracts (especially oil 4), suggested that they did not simply act as chemical scavengers reacting directly with H_2_O_2_ but that there was a real biological action.

**Figure 9:**
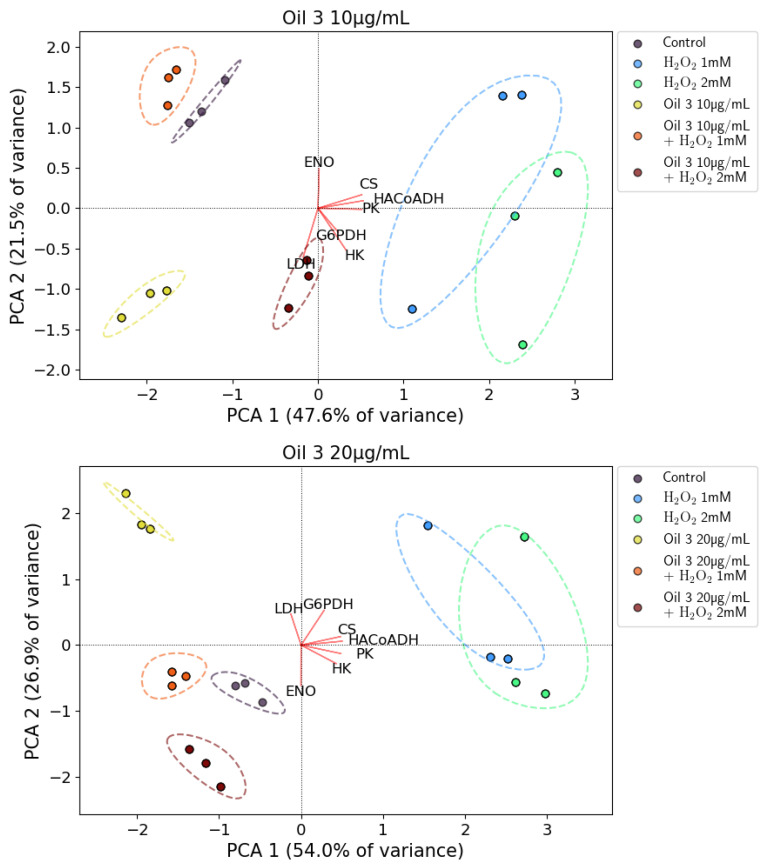
Biplots resulting from the PCA analysis for the two investigated concentrations of the extract of oil 3. Top panel: 10 µg/mL. Bottom panel: 20 µg/mL. Colored circles represent the various samples as specified in the legend (control cells, cells treated with H_2_O_2_ 1 mM and 2 mM, cells treated with 10 µg/mL or 20 µg/mL of the oil 3 extract and cells treated with both the two concentrations of H_2_O_2_ and of the extract of oil 3). The dashed lines of the same colour represent the envelope of each cluster of points to help the reader to view them. The red lines marked with the various enzyme names represent the projections of the relative enzymatic activities on the PCA1 and PCA2 co-ordinates.

**Figure 10:**
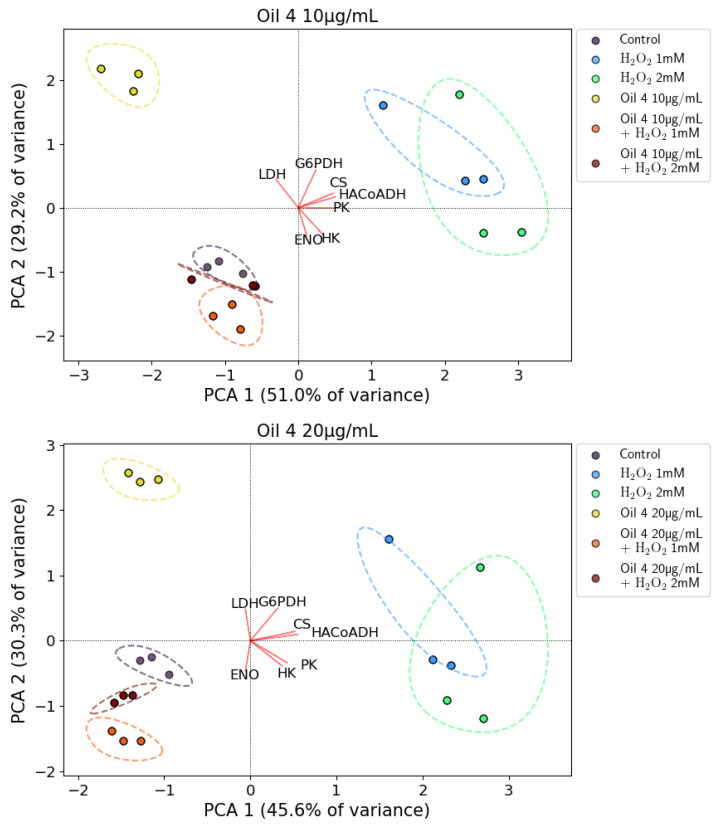
Biplots resulting from the PCA analysis for the two investigated concentrations of the extract of oil 4. Top panel: 10 µg/mL. Bottom panel: 20 µg/mL. Colored circles represent the various samples as specified in the legend (control cells, cells treated with H_2_O_2_ 1 mM and 2 mM, cells treated with 10 µg/mL or 20 µg/mL of the oil 4 extract and cells treated with both the two concentrations of H_2_O_2_ and of the extract of oil 4). The dashed lines of the same colour represent the envelope of each cluster of points to help the reader to view them. The red lines marked with the various enzyme names represent the projections of the relative enzymatic activities on the PCA1 and PCA2 co-ordinates.

The results of the PCA analysis for oil 3 and oil 4 are shown in Figure 9 and Figure 10, respectively. In the top and bottom panel of each figure, the results of the experiments with 10 μg/mL and 20 μg/mL concentrations of both oils are reported, respectively. In each figure, three points are plotted for each sample and these points represent the average result of each of three experiments performed in triplicate. 

Respectively, for the experiments with oil 3 at 10 μg/mL, oil 3 at 20 μg/mL, oil 4 at 10 μg/mL and oil 4 at 20 μg/mL, the first two principal components explained 60.1% of the total variance (PCA1 accounting for 47.6% and PCA2 for 21.5%), 80.9% (54.0% PCA1, 26.9% PCA2), 80.2% (51.0% PCA1, 29.2% PCA2) and 75.9% (45.6% PCA1, 30.3% PCA2), respectively.

Figure 9 and Figure 10 show that the various data samples are well clustered and located in distinct regions of the biplots. The arrangement of the various clusters on the biplot plane confirms the framework of the enzymatic activities discussed above, indicating that the various samples have a well-defined and distinct behavior. 

First of all, the clusters representing pretreatment with both the concentrations of oil 3 and oil 4 are located in the second (oil 3, 20 μg/mL, oil 4, 10 μg/mL and oil 4, 20 μg/mL) and third quadrant (oil 3, 10 μg/mL) of the biplot and are well distinct from the clusters representing the oxidative stress induced with H_2_O_2_ 1 mM and 2 mM that are located in the first/fourth quadrant of the biplot (positive PCA1 axis). This indicates that both the pretreatments with oils and the stimulus with H_2_O_2_ induce a significative increase in some enzymatic activities but the overall framework is different, indicating that those activity changes have a different origin.

The clusters representing the pretreatments with oils together with the H_2_O_2_ stimulus are all located near the control clusters (second quadrant of the biplot for oil 3 at 10 μg/mL and third quadrant for oil 3 at 20 μg/mL, oil 4 at 10 μg/mL and oil 4 at 20 μg/mL). Only the oil 3 at 10 μg/mL + H_2_O_2_ 2 mM cluster is located far from the control cluster and noticeably in a position closer to the H_2_O_2_ clusters.

This picture indicates clearly that pretreatment with the oils can counteract oxidative stress, bringing back the enzymatic activities framework from the one characteristic of the H_2_O_2_-induced oxidative stress to one characteristic of control. As discussed above, this effect is less pronounced for the lower oil 3 concentration that is not capable of counteracting the oxidative stress induced by the higher H_2_O_2_ concentration.

The results discussed above are in agreement with the data presented in a recent work, where the olive pomace (pâté) phytocomplex was tested on the same cell model (AGS) as a countermeasure against oxidative stress induced by H_2_O_2_ [27]. From the chemical point of view, even if pâté and virgin olive oil originated from the same fruit, the phenolic composition of the two phytocomplexes is different. In particular, the composition of the phenolic group in EVOO and pâté is strongly determined by the malaxation phase, during which the secoiridoid compounds are enzymatically transformed into non-glycosylated phenols. Furthermore, the lipid phase concentrates only the most lipophilic molecules, while the more polar ones are retained by the by-product pâté. The differences in the phenolic phytocomplex composition of olive pâté and extra virgin olive oil justify the application of a similar investigation approach in both the manuscripts. 

## 4. Conclusions

This work evaluated for the first time the effect of phenolic extracts obtained from different monovarietal extra virgin olive oils on AGS cells. Among the five studied EVOOs, the two characterized by the greatest differences between their phenolic content were chosen to study the biological effects on cells by evaluating the enzymatic activity of several key enzymes of cellular metabolism.

The results indicate that these phytocomplexes, at the investigated concentrations, do not induce ROS production in gastric cells and are an effective countermeasure to a general oxidative stress. This is a very important aspect in the context of gastrointestinal diseases. Interestingly, the studied phytocomplexes showed the ability to protect cells from oxidative damage but with a different efficacy, probably due to the differences in their phenolic pattern. However, both the secoiridoid derivatives of oleuropein and ligstroside were assessed to contribute to the observed effects. The results of the study suggest that EVOOs with a medium to high concentration of phenols can exert protective effects on the AGS cell regardless of the specific pattern of the sample.

Future developments of this work will concern both the enlargement of the number of the oil samples to investigate and a deep study of the mechanisms underlying their antioxidant action.

## Figures and Tables

**Table 1:** Phenolic concentration in the five monocultivar EVOOs; data from both the IOC official method before hydrolysis and the acid hydrolysis method were expressed in mg/kg of EVOO (mean ± SD from three replicates).

Monocultivar EVOO	Hydroxytyrosol before Hydrolysis	Hydroxytyrosol after Hydrolysis	Tyrosol before Hydrolysis	Tyrosol after Hydrolysis	Total phenols before Hydrolysis	Tyrosol + Hydroxytyrosol after Hydrolysis
	mg/kg	mg/kg	mg/kg	mg/kg	mg/kg	mg/kg
Frantoio (oil 1)	nd	153.8 ± 4.4	nd	134.1 ± 4.0	436.2 ± 27.9	287.9 ± 8.3
Leccino (oil 2)	nd	135.2 ± 3.8	nd	118.3 ± 3.5	367.0 ± 23.5	253.5 ± 7.3
Favolosa (oil 3)	nd	170.7 ± 4.8	nd	149.1 ± 4.4	509.0 ± 32.6	319.8 ± 9.3
Coratina (oil 4)	2.5 ± 0.2	258.5 ± 7.3	3.0 ± 0.2	357.7 ± 10.6	746.4 ± 47.8	616.3 ± 17.9
Tonda Iblea (oil 5)	nd	57.1 ± 1.6	4.1 ± 0.3	109.8 ± 3.3	248.4 ± 15.9	166.9 ± 4.9

**Table 2:** Data on the composition of the five extracts: amount of phenolic dry extract (DE) for 100 mL of EVOOs, amount of total phenolic compounds expressed on DE and ratio between hydroxytyrosol/tyrosol determined after acidic hydrolysis.

EVOO Source of DE	Yelds (mg DE per 100 g EVOO)	Total Phenols mg/g DE	Hydroxytyrosol/Tyrosol after Hydrolysis
Frantoio (oil 1)	100.2	435.1	1.15
Leccino (oil 2)	73.6	498.6	1.14
Favolosa (oil 3)	100.4	507.0	1.14
Coratina (oil 4)	162.2	460.2	0.72
Tonda Iblea (oil 5)	69.8	355.8	0.52

**Table 3:** Closure velocity and closure time of the scratch after treatment with the extracts of oil 3 and oil 4.

	Scratch Closure Velocity (µm/h)	Scratch Closure Time (h)
Control	14.2 ± 1.2	33.0 ± 3.2
Oil 3	3.7 ± 1.2	128.1 ± 41.2
Oil 4	4.7 ± 1.0	87.2 ± 20.2

## Data Availability

Not applicable.
